# Investigation of the Effect of Camellia Sinensis Essence Cream on Skin Burns

**DOI:** 10.3390/life15020176

**Published:** 2025-01-25

**Authors:** Tolga Mercantepe, Adnan Yilmaz, Atilla Topcu, Ali Bilgin, Levent Tumkaya, Filiz Mercantepe

**Affiliations:** 1 Department of Histology and Embryology, Faculty of Medicine, Recep Tayyip Erdogan University, 53010 Rize, Türkiye; 2 Department of Biochemistry, Faculty of Medicine, Recep Tayyip Erdogan University, 53010 Rize, Türkiye; adnan.yilmaz@erdogan.edu.tr; 3 Department of Pharmacology, Faculty of Medicine, Recep Tayyip Erdogan University, 53010 Rize, Türkiye; atilla.topcu@erdogan.edu.tr; 4 Department of Biotechnology, Faculty of Engineering, Samsun University, 55000 Samsun, Türkiye; ali.bilgin@samsun.edu.tr; 5 Department of Histology and Embryology, Faculty of Medicine, On Dokuz Mayıs University, 55270 Samsun, Türkiye; levent.tumkaya@omu.edu.tr; 6 Department of Endocrinology and Metabolism, Faculty of Medicine, Recep Tayyip Erdogan University, 53010 Rize, Türkiye; filiz.mercantepe@saglik.gov.tr

**Keywords:** burn injuries, Camellia sinensis, oxidative stress, collagen synthesis, wound healing

## Abstract

**Background/Objective:** Burn injuries are among the most common causes of trauma globally, affecting millions annually. Current treatments often rely on topical agents, but alternatives to synthetic formulations are increasingly sought due to safety and efficacy concerns. This study aimed to evaluate the therapeutic effects of a cream containing Camellia sinensis (white tea) extract on third-degree burn-induced skin lesions in a rat model. **Methods:** Thirty-two male Sprague-Dawley rats were randomized into four groups: control, Burn only, Burn + Camellia sinensis extract, and Burn + Camellia sinensis cream. Skin biopsies were evaluated using histopathological, immunohistochemical, and biochemical methods. Malondialdehyde (MDA) and glutathione (GSH) levels were measured to assess oxidative stress, while histological damage and immunoreactivity for collagen I, collagen III, NF-kβ/p65, TNF-alfa, 8-OhDG, and caspase-3 were analyzed. **Results:** The Camellia sinensis cream significantly reduced MDA levels and increased GSH levels compared to the burn-only group (*p* < 0.001). Histological analysis revealed enhanced epidermal regeneration and reduced dermal damage. The immunohistochemical findings demonstrated reduced NF-kβ/p65, TNF-alfa, 8-OhDG, caspase-3, collagen I, and collagen III immunopositivity in the cream-treated group (*p* < 0.001). **Conclusions:** Camellia sinensis cream demonstrated significant protective and reparative effects on burn-induced skin damage, suggesting its potential as a natural, effective, and safe alternative for burn management.

## 1. Introduction

Dermal burns constitute a specific category of injury that arises from environmental influences, including elevated temperatures, extreme cold, electrical currents, friction, radiation, or chemical exposure, leading to a compromise in the structural integrity of the skin [1]. The majority of burn incidents occur as a result of domestic mishaps or exposure to solar radiation. According to the World Health Organization (WHO), over 10 million individuals globally are hospitalized annually due to burn-related injuries [2]. While first and second-degree burns typically undergo healing without the formation of scars within a timeframe of 2–3 weeks, third-degree burns involve tissue damage even below the epidermis and sometimes damage to muscles, frequently resulting in scarring and functional impairment, necessitating surgical intervention [3]. For more than half of the burn cases presented in clinical settings, topical ointments serve as the primary modality of treatment, offering a cost-effective and non-invasive alternative [4,5]. Conventional topical formulations encompass anti-inflammatory agents such as zinc oxide, antibiotics including bacitracin, and heavy metal-based compounds such as silver sulfadiazine [6]. Nonetheless, these therapeutic approaches often exhibit limitations, including inadequate efficacy, prolonged healing durations (ranging from 12 to 28 weeks), and safety concerns associated with synthetic constituents [6]. Nevertheless, investigations have been conducted on therapeutic agents derived entirely from organic plant extracts, including Allium cepa, Aloe vera, Santalum album, and Acer palmatum [7,8,9]. These organic formulations are designed to enhance wound healing by augmenting the density of collagen and reticular fibers within connective tissues and by stimulating mitotic activity in the basal layer of the epidermis (stratum germinativum) [7,8,9]. In this regard, research has indicated that plant-derived flavonoids and catechins (EG), particularly epigallocatechin gallate (EGCG), exert a notable influence on the proliferation of collagen and reticular fibrils within subcutaneous connective tissue [10,11]. While earlier investigations indicated that EGCG was predominantly present in green tea, more recent studies have revealed its higher concentration in white tea [12].

Burn-induced dermal injury is typified by a cascade of inflammatory responses, oxidative stress, and mitochondrial impairment, which collectively hinder the process of tissue repair [13]. Prior investigations have demonstrated that catechins derived from green tea mitigate tissue injury through the downregulation of pro-inflammatory mediators, the enhancement of anabolic signaling pathways, and the regulation of microRNA expression [14,15]. Furthermore, these compounds promote protein synthesis and mitochondrial energy metabolism while attenuating age-related deleterious effects [16]. EGCG has been particularly highlighted for its role in facilitating the development of collagen fibers, thus underscoring its therapeutic potential in the realm of cutaneous repair.

The current methodologies for the treatment of burns, which encompass debridement, fasciotomy, grafting, and the application of synthetic coatings, tend to be both financially burdensome and labor-intensive, thereby imposing considerable strains on healthcare systems as well as on patients [17,18]. Moreover, existing topical formulations frequently fall short of delivering holistic healing outcomes, thereby accentuating the pressing need for innovative, efficacious, and safe therapeutic alternatives.

This study aims to explore the therapeutic efficacy of a cream formulated from white tea for the management of burn wounds. The principal objective is to create a cost-effective, user-friendly cream that accelerates the healing process, minimizes adverse effects, and serves as an environmentally sustainable alternative. Additionally, by leveraging the capacity of white tea constituents to enhance collagen fiber density, the research seeks to evaluate its potential as an anti-aging formulation capable of diminishing wrinkles and enhancing overall dermal quality.

## 2. Materials and Methods

### 2.1. Animal Study

A total of 32 male Sprague-Dawley rats, aged 4 months and weighing between 250 and 350 g, were incorporated into the experimental investigation. The subjects were housed in standard laboratory cages under controlled environmental conditions (20–26 °C, 50–70% relative humidity, 12 air exchanges per hour, and a 12 h light–dark cycle), with unrestricted access to food and water. All procedures involving the animals were conducted in accordance with the ethical principles delineated in the National Research Council’s Guide for the Care and Use of Laboratory Animals. Ethical approval for the investigation was granted by the Recep Tayyip Erdoğan University Animal Experiments Local Ethics Committee (approval number: 2020/30; approval date: 16 July 2020).

### 2.2. Application of Burn Model

A total of 32 rats were randomly divided into four groups with similar average weights, and each group consisted of eight rats. All surgical procedures were performed under sterile conditions. The burn model and surgical procedures were performed under 50/10 mg/kg ketamine/xylazine anesthesia. The burn model was implemented in accordance with a modified iteration of the methodologies previously delineated by Vorauer et al. and Alemdaroğlu et al., with modifications guided by our prior experimental insights [19,20,21,22,23]. A 2 cm diameter metal hollow cylinder was placed on the shaved area on the rat’s back skin and boiling hot water (94 ± 1 °C) was poured into the cylinder and held for 17 s. Vorauer et al. employed water at a temperature of 75 °C for a duration of 8 s on rat subjects, yielding second-degree burns across an area measuring 21 cm^2^. In the current investigation, informed by observations from antecedent experiments, we utilized water at 94 °C for a period of 17 s within a cylindrical area of 2 cm in diameter to generate more uniform and reproducible burn wounds that more accurately aligned with our experimental aims. Likewise, while Alemdaroğlu et al. utilized a cylindrical bar with a radius of 1 cm and applied water at 94 °C for 15 s, we modified the cylinder dimensions to a diameter of 2 cm and extended the duration to 17 s. These modifications were instituted to guarantee consistent burn depth and area across all samples. By leveraging insights derived from our earlier work, we enhanced the reproducibility and standardization of burn injuries under the designated experimental conditions, thereby augmenting the originality and applicability of our methodological approach.

The study groups were designed as described below:

Group 1 (*n* = 8): Healthy control group (C); no external intervention was performed.

Group 2 (*n* = 8): Burn group (Burn).

Group 3 (*n* = 8): Burn + Camelia sinensis extract (Burn + CsE); a 2 cm diameter hollow metal cylinder was placed on the shaved area and boiling hot water (94 ± 1 °C) was poured into the cylinder and held for 17 s. Camelia sinensis extract was applied to the burn area for 14 days starting immediately after the burn.

Group 4 (*n* = 8): Burn + Camelia sinensis containing cream (Burn + Cream), a 2 cm diameter hollow metal cylinder was placed on the shaved area and boiling hot water (94 ± 1 °C) was poured into the cylinder and held for 17 s. Camelia sinensis-containing cream was applied for 14 days starting immediately after the burn.

In order to assess the phenomenon of wound contraction, systematic evaluations of alterations in the wound area were conducted. The percentage of wound contraction was determined on the specified days utilizing the subsequent formula [24]:

% Wound Contraction = (Initial Wound Size) − (Final Wound Size)/(Initial Wound Size) × 100.

Metamizole sodium 25 mg/kg i.m. was applied to all the groups with burns for analgesia. Fourteen days after the beginning of the experiment, the rats were euthanized by giving high doses of anesthetic. Some of the skin tissue samples of the rats were stored at −80 °C for use in biochemical studies without breaking the cold chain until the day of the laboratory tests, while the other parts were preserved in 10% neutral formalin for histopathological analyses.

### 2.3. Preparation of White Tea (Camellia Sinensis) Extract

The white tea (WT) specimens utilized in this research were procured from the young shoots of white tea cultivated in the Rize province by the ÇAYKUR General Directorate during the vernal season of 2020.

Three grams of white tea were accurately measured, and one hundred milliliters of previously boiled water was introduced. Following this, the mixture was allowed to steep for a duration of ten minutes under a lid on a magnetic stirrer. Subsequently, the infusion was subjected to filtration using filter paper and was allowed to cool to room temperature while remaining covered. The preparation of this mixture was conducted afresh daily.

### 2.4. Preparation of White Tea (Camellia Sinensis) Cream

Initially, the extraction of white tea was conducted in a manner identical to the previously delineated methodology. Camellia sinensis cream has been formulated to increase the stability of the CsE extract and ensure a longer-lasting effect on the skin. The cream formulation contains a 3% concentration of CsE. The cream is prepared with stabilizing and emulsifying agents (glycerin, stearic acid, and emulsifiers) in a water-based carrier. Additionally, natural oils and vitamins (olive oil, vitamin E) have been included to increase the moisturizing effect of the cream formulation and to strengthen the skin barrier. The procedure for the fabrication of a 3% white tea extract cream was executed within the Research and Development Laboratory of the Faculty of Medicine at Recep Tayyip Erdoğan University (Supplement Table S1).

### 2.5. Biochemical Analyses

#### 2.5.1. Tissue Homogenization

A 20 mM solution of sodium phosphate in 1 L, along with 140 mM potassium chloride, was formulated to achieve a pH of 7.4. Subsequently, 1 mL of homogenization solution was incorporated into 100 mg of tissue, which was then homogenized utilizing a skin tissue homogenizer (QIAGEN TissueLyser II), followed by centrifugation at 800× g for a duration of 10 min at a temperature of 4 °C. The total thiol (TT) content and thiobarbituric acid reactive substances (TBARS) were assessed using the resultant supernatant.

#### 2.5.2. Malondialdehyde (MDA) Analysis Procedure (TBARS Assay)

The quantification of TBARS was executed in accordance with the methodology established by Ohkawa et al. [25]. A solution was formulated comprising 200 µL of tissue supernatant; 50 µL of 8.1% SDS (sodium dodecyl sulfate); 375 µL of 20% acetic acid (*v*/*v*) at a pH of 3.5; and 375 µL of 0.8% thiobarbituric acid (TBA). The resultant mixture was subjected to vortexing, and the ensuing reaction was allowed to incubate in a boiling water bath for a duration of 1 h. Subsequent to the incubation period, the mixture was cooled in ice water for 5 min and then centrifuged at a force of 750× g for 10 min. The resulting pink chromophore was quantified using a spectrophotometer at a wavelength of 532 nm. The outcomes were expressed as nmol/mg of protein.

#### 2.5.3. Total Tiol (GSH) Analyses

Following the addition of 1000 µL of 3 M Na_2_HPO_4_ and 250 µL of DTNB to 250 µL of supernatant (comprising 4 mg of DTNB dissolved in 10 mL of a 1% sodium citrate solution) and subsequent vortex mixing, the absorbance was quantified at a wavelength of 412 nm [26]. The results were ascertained utilizing the constructed standard curve of reduced glutathione ranging from 1000 µM to 62.5 µM and expressed in terms of nmol/mg of protein.

### 2.6. Histopathological Analyses

Skin tissues were sectioned into uniform 1.5 cm^3^ fragments and subsequently preserved in a 10% neutral formalin solution (Sigma-Aldrich, Steinheim, Germany) for a duration of 24 h. Following the fixation period, these specimens were transferred into tissue-processing cassettes and subjected to a sequential series of increasing concentrations of ethyl alcohol (50%, 60%, 70%, 80%, 96%, and 100% [twice]) utilizing a tissue processing apparatus (ThermoScientific™ Citadel 2000, London, UK). In the subsequent phase, the tissues underwent a clearing process with a series of xylene (xylol). The specimens were then encapsulated in paraffin blocks using tissue embedding cassettes equipped with metal molds in a paraffin embedding apparatus (Leica EG 1150 H, Wetzlar, Germany). Sections with a thickness of 4–5 µm were excised from these blocks employing a rotary microtome (Leica RM2255, Wetzlar, Germany) and were subsequently stained with hematoxylin and eosin G (H&E, Merck GmbH, Darmstadt, Germany) utilizing a staining device (Leica ST5020, Wetzlar, Germany).

### 2.7. Immunohistochemical Analyses

In the context of immunohistochemical (IHC) analyses, the following primary antibodies were used: cleaved caspase-3 (ab4051, Abcam, Cambridge, UK), collagen type I (collagen Ia3, ab34710, Abcam, Cambridge, UK), collagen type III (collagen IIIa1, ab23445), TNF alpha (ab220210, Abcam, Cambridge, UK), NF-Kb/p65 (ab32536, Abcam, Cambridge, UK), and 8-hidroksi-2p-deoksiguanozin (8-OHdG) primary antibody (ab285254, Abcam, Cambridge, UK). Additionally, secondary antibodies that exhibited compatibility with the aforementioned primary antibodies (Goat Anti-Rabbit IgG H&L (HRP), ab205718, Abcam, Cambridge, UK) were also utilized. The tissue sections, which were affixed to positively charged slides, underwent staining via a fully automated immunohistochemistry staining apparatus (Bond Max III, Leica Biosystems, Nussloch, Germany).

In the context of immunohistochemical (IHC) investigations, chromogen 3,3′-Diaminobenzidine (DAB) (Bond Polymer Refine Detection, LBS Deutschland GmbH, DS9800, Newcastle Upon Tyne, UK) was employed to elucidate the immunopositivity levels of the target proteins. The application of DAB resulted in a brown hue in regions exhibiting immunopositivity, thereby indicating the presence and concentration of the proteins under examination. For the purpose of counterstaining cellular nuclei, Harris hematoxylin was utilized, resulting in nuclei being stained a distinctive blue. This process of counterstaining facilitated the delineation of the immunopositivity brown regions from those that were negative. In the resultant imagery, the brown coloration signifies the positive immunoreactivity of the target proteins, whereas the blue coloration denotes the presence of cell nuclei.

### 2.8. Semi-Quantitative Analyses

Histopathological evaluations of H&E-stained dermal tissue specimens were systematically scored as delineated in Table 1, employing the Skin Histopathological Damage Score (SHDS) established by Jeschke et al. and Gibson et al., as well as the modified version of the Skin Damage Score proposed [27,28]. In this current investigation, the assessment for quantifying immune-positive cells within sections subjected to IHC was executed according to the following criteria: 0 = absent =≤ 5%, 1 = mild = 6–25%, 2 = moderate = 26–50%, 3 = severe => 50%. Semiquantitative analyses were conducted by two histopathologists who were blinded to the respective study groups, with the evaluation encompassing 20 distinct fields randomly selected from the sections of each rat, utilizing a ×20 objective magnification.

### 2.9. Statistical Analyses

Data derived from various analyses were subjected to statistical evaluation utilizing SPSS 18.0 software (IBM Corp., Armonk, NY, USA). The parametric characteristics of the histological data acquired in our investigation were assessed through the implementation of Shapiro–Wilk, Q–Q plot, skewness–kurtosis value, and Levene’s tests. In the aftermath of the normality assessments, data extracted from the semi-quantitative analysis were computed as the mean alongside the 25th and 75th interquartile ranges. Subsequently, intergroup differences were analyzed utilizing the Kruskal–Wallis test followed by Tamhane’s T2 test. A *p*-value of less than 0.05 was deemed statistically significant. Conversely, biochemical data were calculated as mean ± standard deviation for continuous variables exhibiting normal distribution. The analyses were conducted by employing one-way analysis of variance (ANOVA) to assess intergroup differences, followed by a Tukey HSD test (with a significance threshold set at *p*-value <0.05).

## 3. Results

### 3.1. Clinical Evaluation of Wound Closure and Quantitative Measurement of Wound Contraction

**Figure 1:**
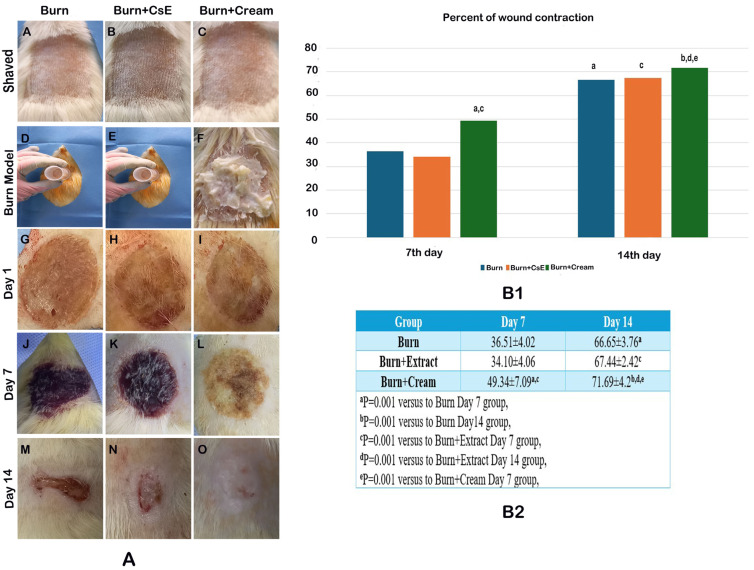
(**A**) Application of burn model and evaluation of wound closure. (**B1**,**B2**) Clinical evaluation of wound healing regarding wound contraction in rats at days 1, 7, and 14.

In Figure 1, the appearances of burn injuries in rats on the 1st, 7th, and 14th days following the implementation of the burn application model are illustrated (Figure 1A); the results of the comparative analysis regarding wound contraction are delineated in both tabular and graphical formats (Figure 1B1,B2). When the Burn, Burn + CsE, and Burn + Cream groups were assessed internally, a statistically significant enhancement in the percentage of wound contraction was observed on the 14th day when juxtaposed with the 7th day following the application of the burn across all three groups. In the comparative analysis among the groups, the wound contraction noted in the Burn + CsE group on the 7th day of the burn model implementation was found to be analogous to that of the Burn group. Conversely, the Burn + Cream group exhibited markedly superior wound contraction when compared to both the Burn and Burn + EsC groups (*p* = 0.001, *p* = 0.001, respectively). Likewise, on the 14th day post-application, the wound contraction documented in the Burn + CsE group was comparable to that of the Burn group. However, the Burn + Cream group again demonstrated statistically significant higher wound contraction in relation to both the Burn and Burn + EsC groups (*p* = 0.001, *p* = 0.001, respectively). These findings elucidate that the application of cream (Burn + Cream group) substantially augmented the wound healing process in contrast to the other groups, particularly on both the 7th and 14th days. Furthermore, the enhancement in wound contraction was notably more pronounced over time within the Burn + Cream group, thereby underscoring its efficacy in facilitating expedited wound closure.

### 3.2. Biochemical Results

The biochemically assessed levels of TBARS (MDA) and TT (GSH) within the skin tissue are presented in Table 2. The tissue MDA concentration was quantified at 10.54 ± 1.11 nmol/g tissue in the healthy group, whereas it was measured at 19.33 ± 0.94 nmol/g tissue in the Burn group, representing a statistically significant elevation in comparison to the control group (*p* < 0.001). Conversely, the MDA concentration exhibited a reduction to 18.63 ± 0.90 nmol/g tissue within the Burn + CsE group; however, this reduction did not reach statistical significance. Conversely, within the Burn + Cream group, MDA levels diminished to 11.32 ± 1.14 nmol/g tissue, and this reduction was determined to be statistically significant when compared to the Burn group (*p* < 0.001).

TT levels were recorded at 8.03 ± 0.93 nmol/g tissue in the control group. Furthermore, TT levels were observed to diminish to 4.77 ± 0.38 nmol/g tissue in the Burn group, a finding that was statistically significant (*p* < 0.001). In contrast, within the Burn + CsE group, this level increased to 4.96 ± 0.86 nmol/g tissue; however, this change did not achieve statistical significance. In the Burn + Cream group, TT levels were quantified at 8.13 ± 1.42 nmol/g tissue, which represented a statistically significant increase when compared to the Burn group (*p* < 0.000).

### 3.3. Histopathological Results

**Figure 2:**
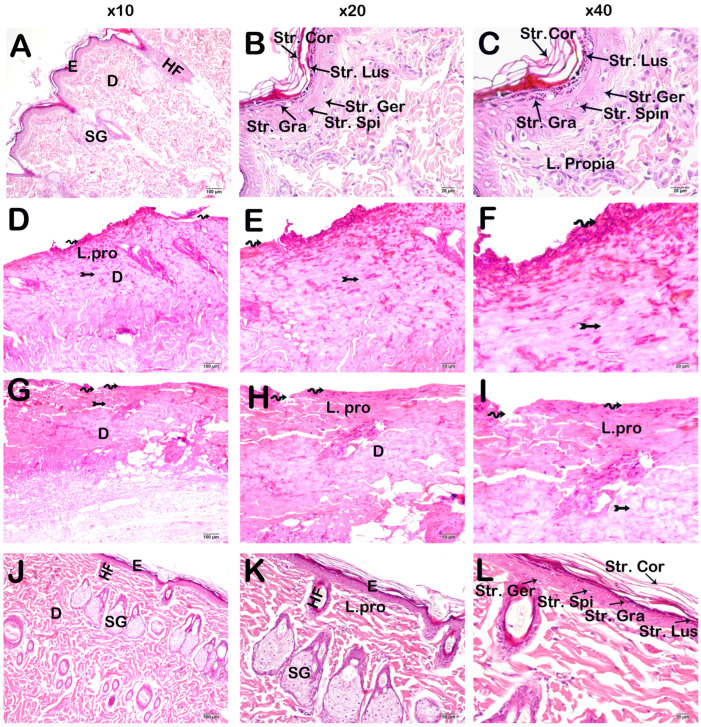
Representative light microscopic image of skin lesions formed in subjects as a result of the Grade III burn model. Stratum germinativum (Str. Ger), Stratum Spinosum (Str. Spi) Stratum Granulosum (Str. Gra), Stratum lucidum (Str. Lus), Stratum Corneum (Str. Cor), Lamina propria (L. Propria), Epidermis (E), Dermis (D), Sebaceous Gland (Y. Gland), Hair Follicle (Hair Fol). (**A**) (×10)-(**B**) (×20)-(**C**) (×40): In the sections of the control group, the epidermis layer consisting of the normal structure of str. germinativum, str. spinosum, str. granulosum, str. lucidum, and str. corneum layers is observed. In addition, it is observed that the lamina propria and dermis (D) layer are of typical structure (SHDS score: 0 (1–0)). (**D**) (×10)-(**E**) (×20)-(**F**) (×40): In the sections of the Burn group, the epidermis layer is completely shed (spiral arrow) and hemorrhage is observed in the lamina propria (tailed arrow) (SHDS score: 4 (4–4)). (**G**) (×10)-(**H**) (×20)-(**I**) (×40): In the sections of the Burn + CsE group, keratinocytes forming the epidermis layer are completely shed (spiral arrow) and hemorrhagic areas in the lamina propria layer (tailed arrow) are observed (SHDS score: 4 (3–4)). (**J**) (×10)-(**K**) (×20)-(**L**) (×40): In the sections belonging to the Burn + Cream group, the epidermis layer consisting of str. germinativum, str. spinosum, str. granulosum, str. lucidum, and str. corneum layers with a normal structure is observed. In addition, it is observed that the lamina propria (L. Propria) and dermis (D) layers are in a healthy structure. Sebaceous glands and hair follicles are present (SHDS score: 1 (1–1)).

The findings derived from the assessment of keratinocyte attrition within the str. germinativum, str. spinosum, str. granulosum, str. lucidum, and str. corneum layers of the epidermis, alongside the evaluation of damage within the dermal layer utilizing the Skin Histopathological Damage Scoring (SHDS) methodology, are shown in Figure 2 and Table 3. In contrast to the control group, which exhibited a structurally intact epidermis, the Burn group demonstrated a complete ablation of all epidermal layers within the affected region, alongside extensive hemorrhagic manifestations and structural compromise in both the dermis and lamina propria layers. Likewise, in the group subjected to treatment with Camellia sinensis extract, a notable loss of keratinocytes within the epidermis and pronounced damage in the dermis layer were observed. Conversely, in the group receiving burn cream formulated with Camellia sinensis, it was discerned that the epidermis, particularly the Malpighian layer encompassing the str. germinativum and str. spinosum layers, retained a typical and well-defined architecture. Furthermore, there was a marked reduction in hemorrhagic regions within the lamina propria and dermis layers, accompanied by the identification of numerous distinct sebaceous glands and hair follicles.

### 3.4. Immunohistochemical Results

**Figure 3:**
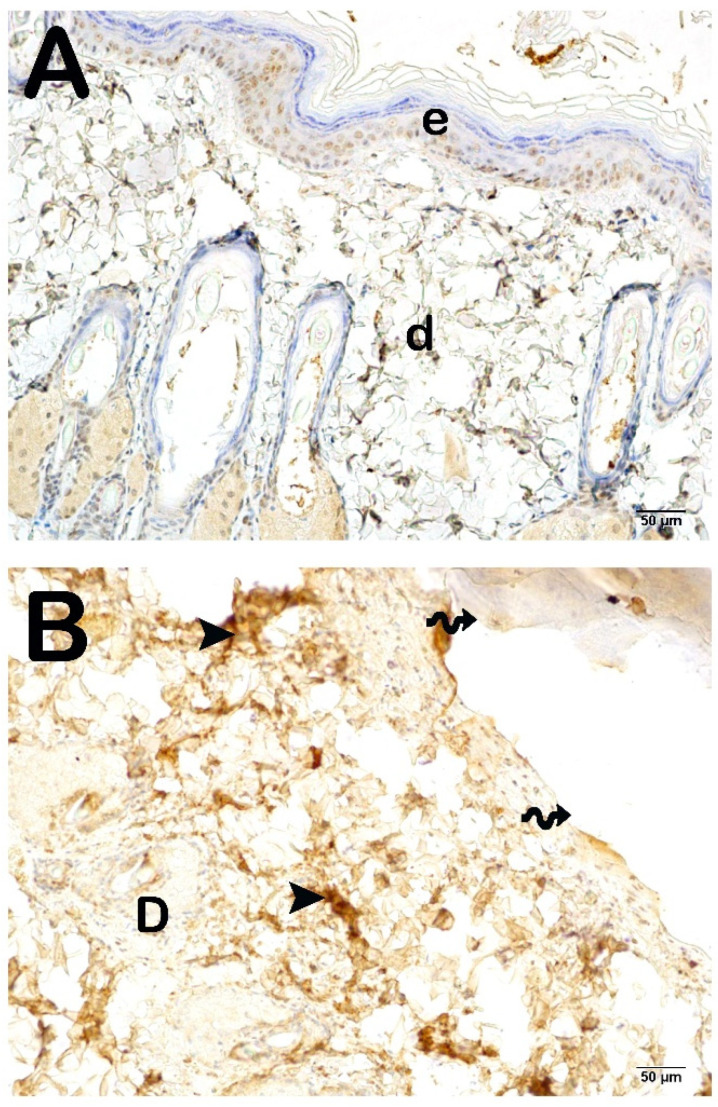

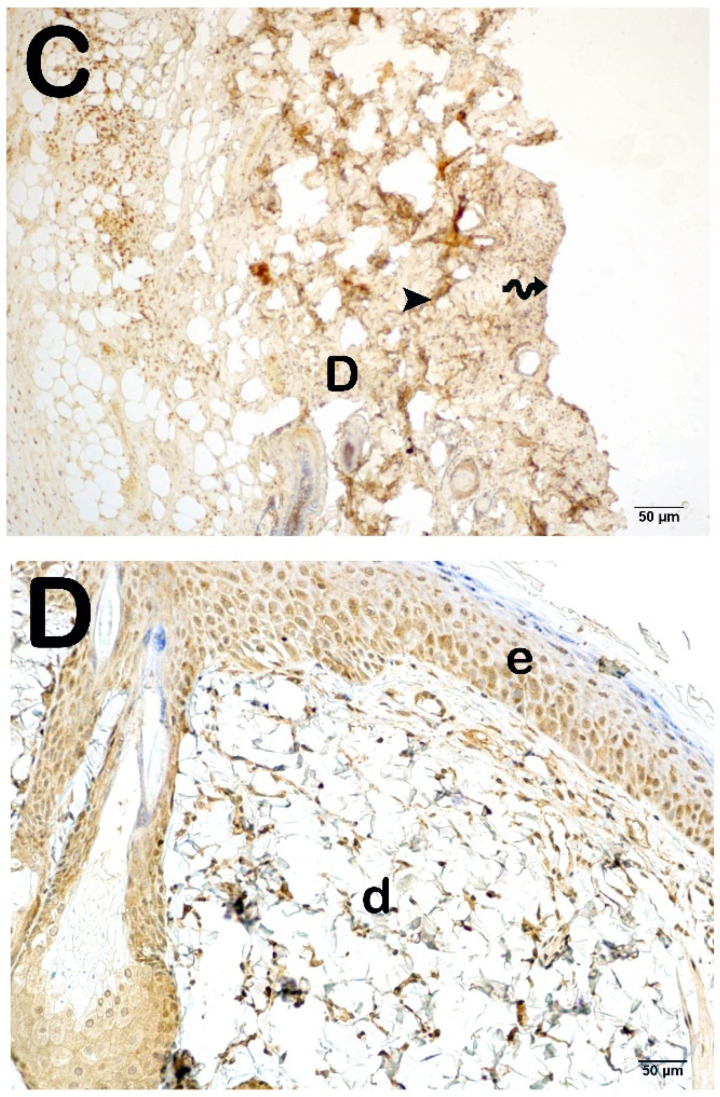
Representative light microscopic image of skin tissue sections incubated with collagen I (Col1A3) primary antibody. Epidermis (E), Dermis (D). (**A**) (×20): Epidermis (E) layer consisting of str. germinativum, str. spinosum, str. granulosum, str. lucidum, and str. corneum layers in the sections of the control group. Lamina propria and dermis (D) layers show slight positivity with collagen 1 primary antibody (collagen I primary antibody positivity score: 0 (0–0)). (**B**) (×20): In the sections belonging to the Burn group, it is observed that the epidermis layer is completely shed (spiral arrow), and the lamina propria and dermis (D) layer show intense positivity for the primary antibody collagen 1 (arrowhead) (collagen I primary antibody positivity score: 3 (3–4)). (**C**) (×20): In the sections of the Burn + CsE group, it is observed that the keratinocytes forming the str. germinativum, str. spinosum, str. granulosum, str. lucidum, and str. corneum are completely shed (spiral arrow). Intense positivity with the collagen 1 primary antibody is observed in the lamina propria and dermis (D) layer (arrowhead) (collagen I primary antibody positivity score: 4 (3–4)). (**D**) (×20): In the Burn + Cream group, normal epidermis (E) and dermis (D) structures are observed. It is observed that the number of cells showing collagen I positivity in the dermis has decreased (collagen I primary antibody positivity score: 1 (1–1)).

**Figure 4:**
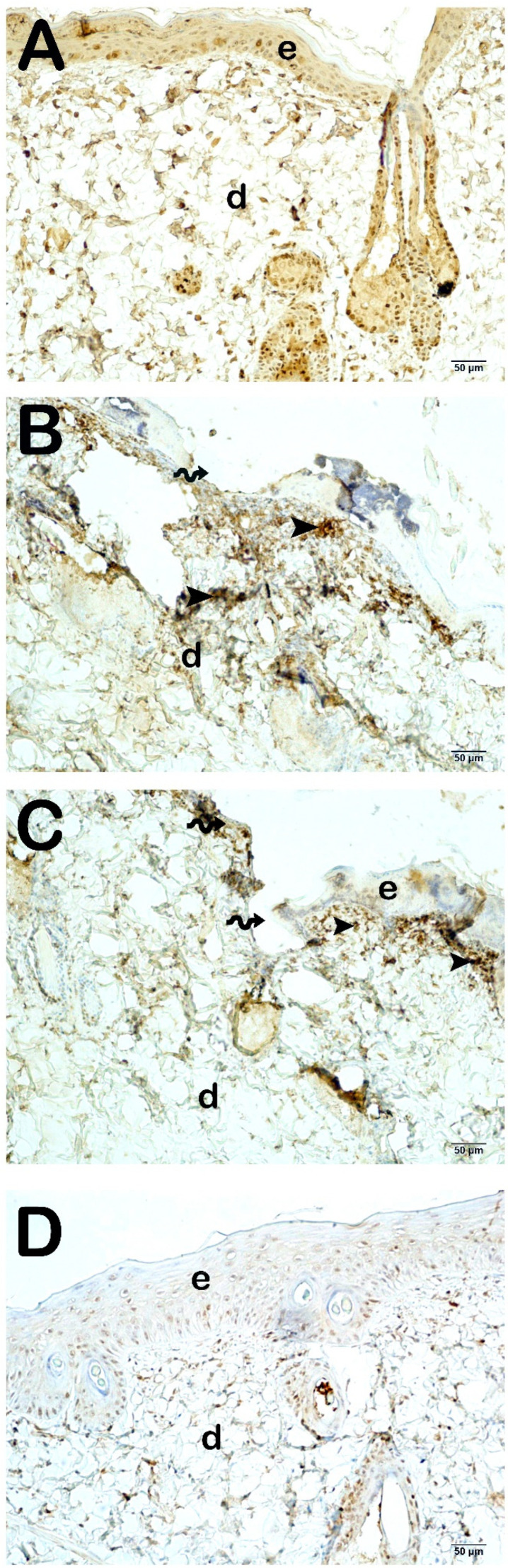
Representative light microscopic image of skin tissue sections incubated with collagen III (Col3A1) primary antibody. Epidermis (e), Dermis (d). (**A**) (×20): In the sections of the control group, the normal structure of the epidermis (E), lamina propria, and dermis (D) layers are observed. It is observed that collagen III primary antibody shows slight positivity (collagen III primary antibody positivity score: 0 (0–0)). (**B**) (×20): In the sections belonging to the Burn group, it is observed that the epidermis layer is completely shed (spiral arrow), and the lamina propria and dermis (D) layers show intense positivity with the collagen III primary antibody (arrowhead) (collagen III primary antibody positivity score: 3 (3–4)). (**C**) (×20): In the sections of the Burn + CsE group, it is observed that the keratinocytes forming str. germinativum, str. spinosum, str. granulosum, str. lucidum, and str. corneum are completely shed (spiral arrow). Intense positivity with the collagen III primary antibody is observed in the lamina propria and dermis (D) layer (arrowhead) (collagen III primary antibody positivity score: 4 (3–4)). (**D**) (×20): In the Burn + Cream group, normal epidermis (E) and dermis (D) structures are observed. It is observed that the number of cells showing collagen III positivity in the dermis decreased (collagen III primary antibody positivity score: 1 (1–1)).

To assess collagen synthesis within the dermal layer, histological sections of the skin were subjected to incubation with primary antibodies targeting collagen type I and collagen type III, subsequently analyzed under a light microscope. The findings are illustrated in Figure 3 and Figure 4 and summarized in Table 4. When compared to the control group, a statistically significant augmentation in the number of cells exhibiting pronounced positivity for collagen type I and collagen type III was identified in the Burn group (*p* < 0.001). Likewise, in the Burn + CsE group, there was an observable increase in collagen type I and type III positivity relative to the control group (*p* < 0.001). Conversely, in the Burn + Cream group, a reduction in the number of cells demonstrating positivity for collagen type I and type III was noted when compared with the Burn group (*p* < 0.001).

**Figure 5:**
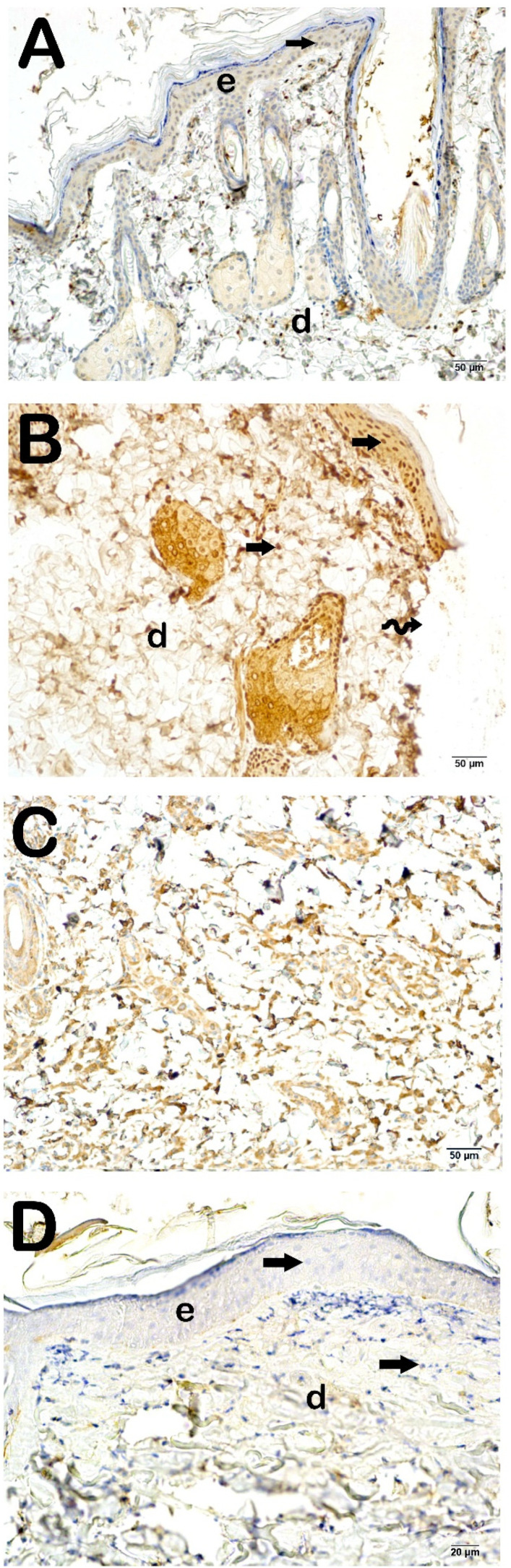
Representative light microscopic image of skin tissue sections incubated with cleaved caspase-3 primary antibody. Epidermis (e), Dermis (d). (**A**) (×20): In sections from the control group, normal structured epidermis (E), lamina propria, and dermis (D) layers are observed (cleaved caspase-3 primary antibody positivity score: 0 (0–0)). (**B**) (×20): In the sections of the Burn group, the epidermis layer is completely shed in the area where the burn lesion is located (spiral arrow), and in the epidermis layer where the lesion is not present, there is intense cleaved caspase-3 positivity in numerous apoptotic keratinocytes (arrowhead) (cleaved caspase-3 primary antibody positivity score: 3 (3–4)). (**C**) (×20): In the sections of the Burn + CsE group, numerous cells showing intense cleaved caspase-3 positivity are observed (arrowhead) (cleaved caspase-3 primary antibody positivity score: 4 (3–4)). (**D**) (×20): D(×20): In the Burn + Cream group, normal epidermis (E) and dermis (D) structures are observed. It is observed that the number of cells showing cleaved caspase-3 positivity in the dermis is decreased (cleaved caspase-3 primary antibody positivity score: 1 (1–1)).

The findings of skin sections incubated with primary antibodies for cleaved caspase-3, aimed at evaluating apoptosis in keratinocytes within the epidermal layer, are presented in Figure 5 and Table 4. In the Burn group, apoptotic keratinocytes exhibiting strong cleaved caspase-3 positivity were identified in the str. granulosum, str. lucidum, and str. corneum layers, with particular prominence in the str. germinativum and str. spinosum layers. Within the Burn group treated with Camellia sinensis extract; a notable presence of intense cleaved caspase-3 positivity was similarly observed in the keratinocytes of the epidermal layer. In contrast, in the group receiving camellia sinensis cream, a decrease in cleaved caspase-3 positivity in keratinocytes was particularly evident in the Malpighian layer (Str. germinativum + Str. spinosum) when compared to the Burn group (*p* < 0.001).

**Figure 6:**
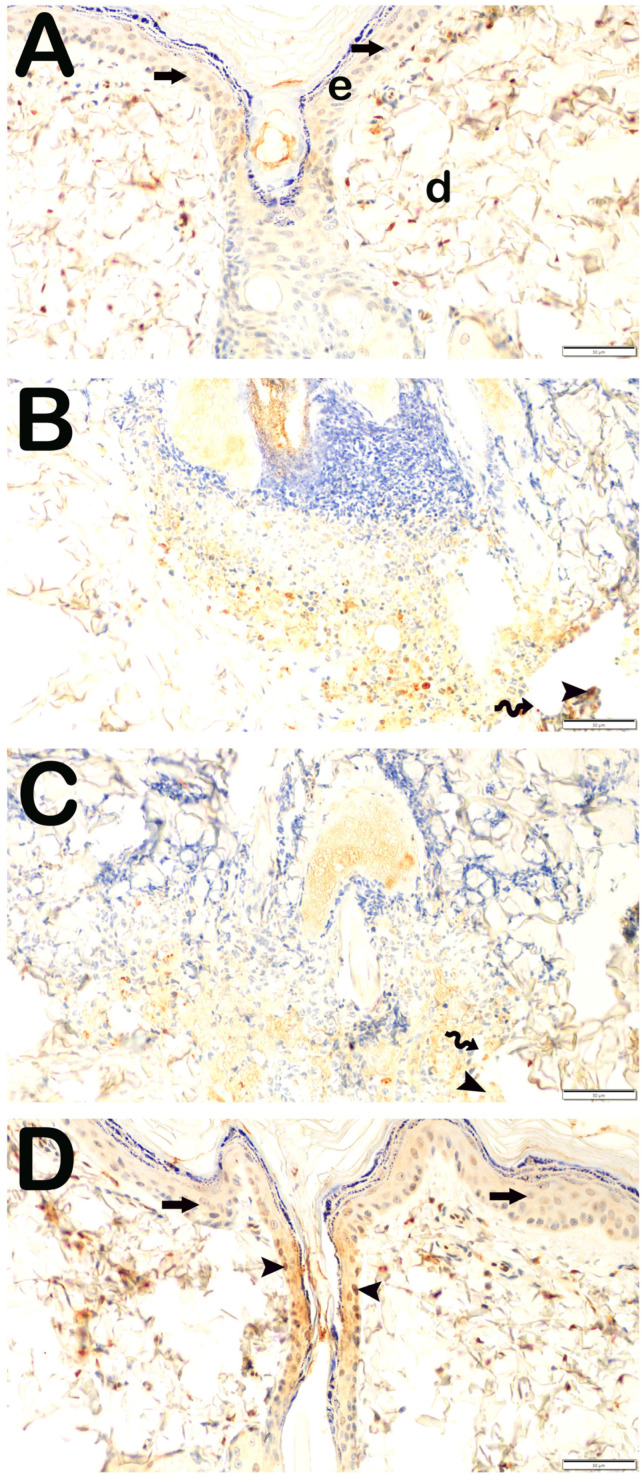
Representative light microscopic image of sections of skin tissue incubated with TNF-α primary antibody. Epidermis (e), Dermis (d). (**A**) (×20): In sections from the control group, normal structured epidermis (E), lamina propria, and dermis (D) layers are observed (TNF-α primary antibody positivity score: 0 (0–0)). (**B**) (×20): In the sections of the Burn group, the epidermis layer is completely shed (spiral arrow) in the area where the burn lesion is located and numerous keratinocytes with intense TNF-α positivity are observed (arrowhead) (TNF-α primary antibody positivity score: 2 (1–2)). (**C**) (×20): In the sections belonging to the Burn + CsE group, numerous cells showing intense TNF-α positivity are observed (arrowhead) (TNF-α primary antibody positivity score: 2 (1–2)). (**D**) (×20): In the Burn + Cream group, normal epidermis (E) and dermis (D) structures are observed. It is observed that the number of cells showing TNF-α positivity in the dermis has decreased (TNF-α primary antibody positivity score: 1 (0–1)).

**Figure 7:**
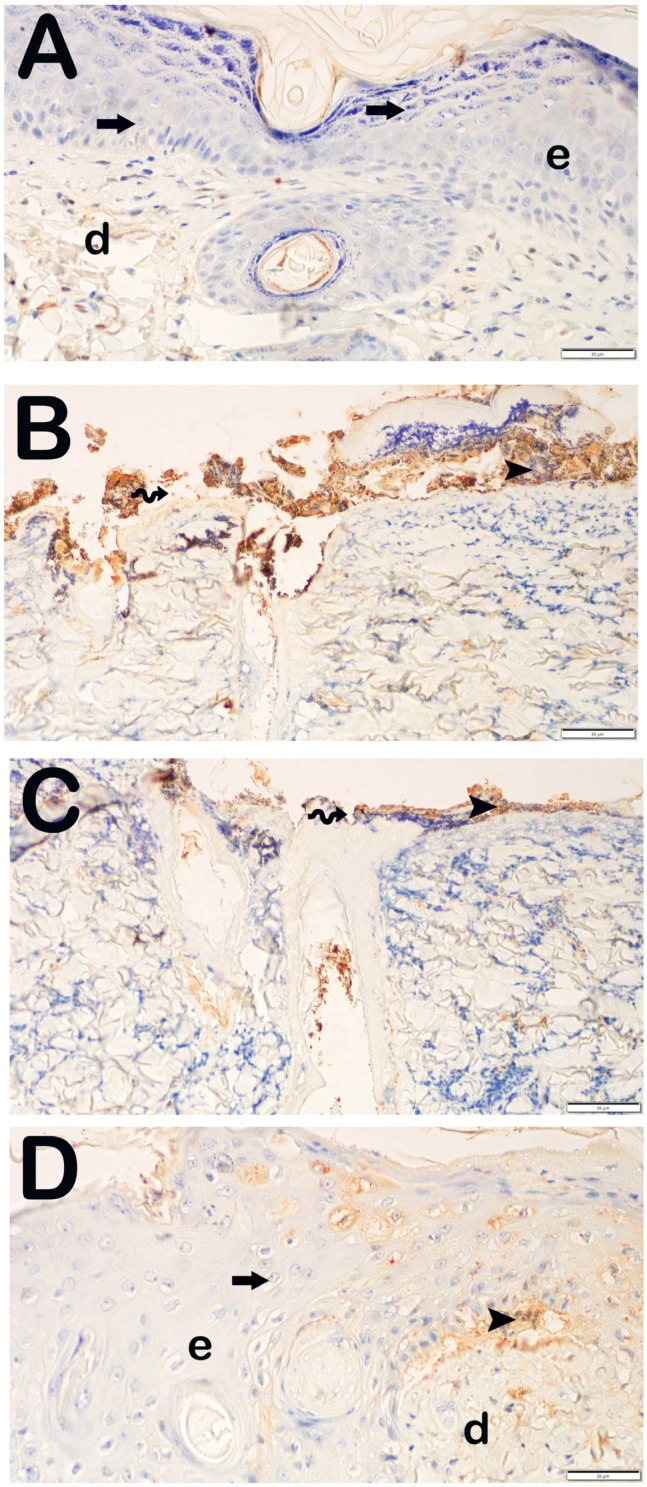
Representative light microscopic images of skin tissue sections incubated with NF-Kβ/p65 primary antibody. Epidermis (e), Dermis (d). (**A**) (×20): In the sections of the control group, it is observed that the cells in the normal structured epidermis (E) and dermis (D) layers are immunonegative (NF-Kβ/p65 primary antibody positivity score: 0 (0–0)). (**B**) (×20): In the sections of the Burn group, the epidermis layer is completely shed in the area of the burn lesion (spiral arrow) and numerous keratinocytes showing intense NF-Kβ/p65 positivity are observed (arrowhead) (NF-Kβ/p65 primary antibody positivity score: 1 (1–2)). (**C**) (×20): In the sections of the Burn + CsE group, numerous cells showing intense NF-Kβ/p65 positivity are observed (arrowhead) (NF-Kβ/p65 primary antibody positivity score: 1 (1–2)). (**D**) (×20): It is observed that the number of cells showing NF-Kβ/p65 positivity decreased in the Burn + Cream group (NF-Kβ/p65 primary antibody positivity score: 0 (0–1)).

Immunohistochemical analyses of dermal tissue specimens utilizing primary antibodies targeting TNF-α and NF-kβ/p65 to assess pro-inflammatory cytokine expression are depicted in Figure 6 and Figure 7 and summarized in Table 4. Within the Burn group, a substantial prevalence of TNF-α and NF-kβ/p65-positive keratinocytes were identified in the str. granulosum, str. lucidum, and str. corneum, with particular prominence noted in the str. germinativum and str. spinosum. Similarly, the Burn + CsE group exhibited pronounced immunoreactivity in keratinocytes concerning TNF-α and NF-kβ/p65 primary antibodies within the epidermal layer. Conversely, in the skin tissue specimens from the Burn + Cream group, a marked reduction in the positivity for TNF-α and NF-kβ/p65 primary antibodies was observed, particularly within the keratinocytes of the Malpighian layer (str. germinativum + str. spinosum) when compared with the Burn group (*p* < 0.001).

**Figure 8:**
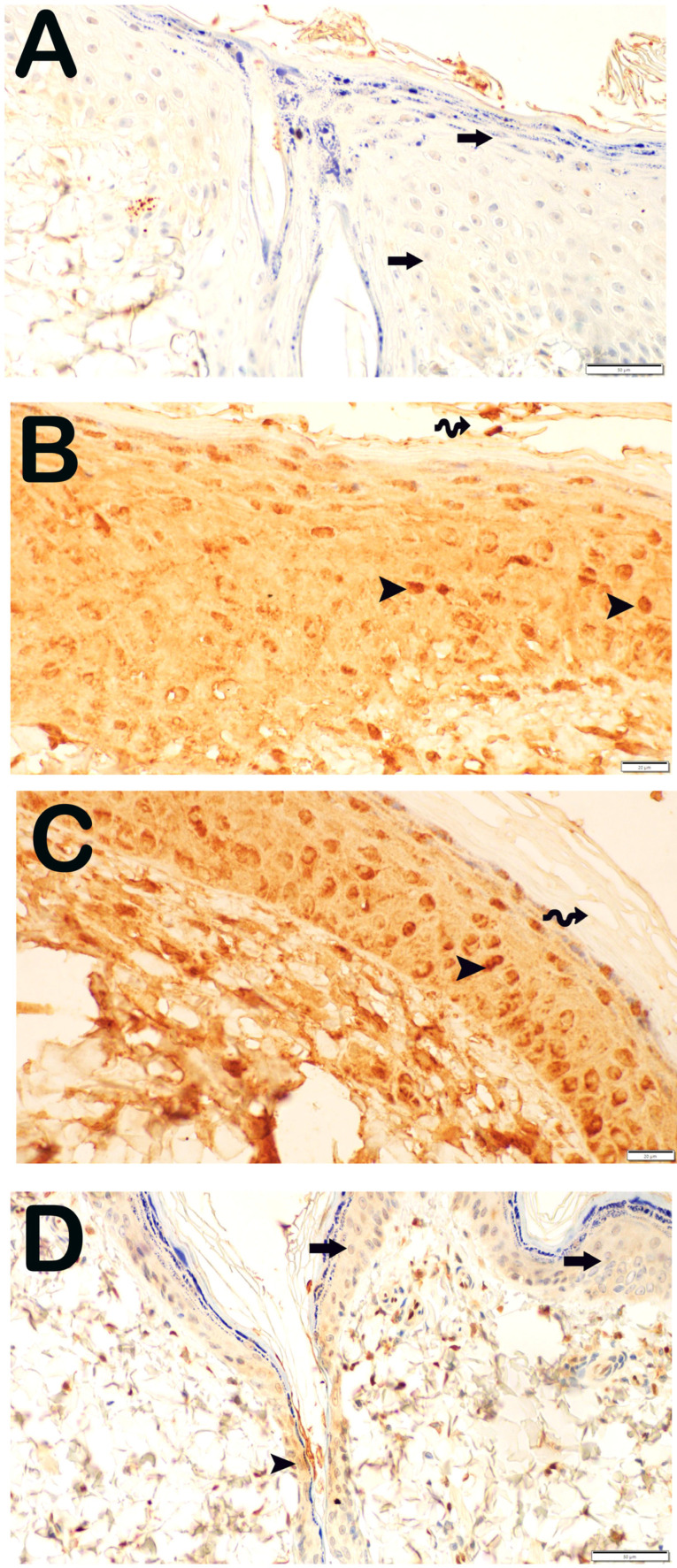
Representative light microscopic image of skin tissue sections incubated with 8-OHdG primary antibody. (**A**) (×20): In sections from the control group, immunonegative cells are observed in terms of 8-OHdG primary antibody (8-OHdG primary antibody positivity score: 0 (0–0)). (**B**) (×20): In the sections of the Burn group, the epidermis layer is completely shed in the area where the burn lesion is located (spiral arrow) and numerous keratinocytes showing intense 8-OHdG positivity are observed (arrowhead) (8-OHdG primary antibody positivity score: 3 (2–3)). (**C**) (×20): In the sections of the Burn + CsE group, numerous cells showing intense immuno-positivity are observed (arrowhead) (8-OHdG primary antibody positivity score: 3 (2–3)). (**D**) (×20): It is observed that the number of cells showing 8-OHdG positivity decreased in the Burn + Cream group (8-OHdG primary antibody positivity score: 0 (0–1)).

The immunohistochemical findings of the dermal tissue sections that were incubated with the 8-OHdG primary antibody to evaluate oxidative DNA damage are illustrated in Figure 8 and detailed in Table 4. A significant number of keratinocytes exhibiting strong 8-OHdG positivity were noted within the epidermal layers of the Burn group. In the skin tissue sections from the group treated with Camellia Sinensis extract, significant 8-OHdG positivity was also detected in the keratinocytes located in the epidermal layer. In contrast, a notable decrease in 8-OHdG positivity among keratinocytes was recorded in the skin tissue sections from the Burn + Camellia Sinensis cream application group in comparison to the Burn group (*p* < 0.001).

## 4. Discussion

This research examined the impact of a topical cream formulation containing Camellia sinensis (white tea) extract on dermal burn injuries and the wound-healing process through the application of histopathological, immunohistochemical, and biochemical methodologies. The results of the investigation indicate a prospective function of the cream formulation enriched with white tea extract in facilitating the repair of skin lesions.

The dermal system represents the largest organ within the human anatomy, serving critical functions including protection, thermoregulation, and sensory perception, and comprises three distinct layers: the epidermis, dermis, and hypodermis [29,30]. The epidermis constitutes the outermost stratum of the skin, predominantly formed by cells known as keratinocytes. The dermis, situated beneath the epidermal layer, constitutes the secondary layer of the skin. The principal cellular constituents of the dermis are fibroblasts, which are integral in forming the extracellular matrix that provides structural support to this layer [3,10,29]. The hypodermis represents the deepest layer of the skin and is primarily composed of cells referred to as lipocytes. In investigations concerning burns, the specific agents exerting a direct influence on the epidermis remain largely unidentified; however, numerous studies have been conducted to elucidate their impacts on the dermal layer [3]. The process of skin repair is systemic in nature, entailing a series of stages including hemostasis (coagulation), inflammation (infiltration of mononuclear cells), proliferation (which encompasses epithelialization, fibroplasia, angiogenesis, and the formation of granulation tissue), and maturation (characterized by collagen deposition or tissue remodeling) at the site of injury [9,31,32,33,34]. Each of these four systemic processes observed during skin regeneration in instances of burns exhibits variability influenced by diverse factors such as the etiology, severity, extent of the burn, and the overall health status of the patient. Variations in these processes subsequently result in modifications to burn treatment protocols. The efficacy of the healing process may yield disparate outcomes contingent upon the burn’s severity. Superficial burns typically undergo healing within a fortnight, resulting in minimal scarring. The re-epithelialization of partial-thickness burns is facilitated by the migratory behavior of keratinocytes from the dermal extensions of the skin shortly following the injury. In instances of deeper burns, healing initiates at the periphery rather than centrally due to the imperative for expedited wound closure. The enhancement of early cellular proliferation, which is conducive to rapid burn healing, is mediated by the release of various factors from dendritic cells. Consequently, agents that promote the activity of dendritic cells are being explored as therapeutic interventions to optimize burn wound management [9,31,32,33,34].

The histological findings of this study showed that the cream formulation containing white tea accelerated epidermal healing and provided improvements in the morphological structure of the epidermis. Histopathological analyses revealed that the epithelial renewal was faster and the epidermis gained a more regular structure in the groups treated with the cream containing white tea compared to the control groups. The improvement observed in the thickness and cellular structures of the epidermis suggests that the formulation may create positive changes in the upper layer of the skin. Recent studies have reported that epigallocatechin gallate (EGCG) has positive effects on dendritic cells in the epidermis layer [7,10,35]. In addition, in the study conducted by Hsu et al., EGCG and flavonoids were shown to provide keratinocyte growth in the epidermis layer and reactivate dying old keratinocytes [36]. It has been reported in the literature that EGCG, epigallocatechin (EGC), and flavonoids activate keratinocytes [9,30]. It has been reported that EGCG, catechins (EG), and flavonoids, which are found in high amounts in white tea, have multifaceted positive effects on keratinocytes and dendritic cells in the epidermis layer of the skin, as well as increasing the number of collagen and reticular fibrils in the dermis and hypodermis layers of the skin [9,37,38]. In this present study, the increase observed in the thickness of the epidermis layer suggests that the cream formulation may promote keratinocyte proliferation. In addition, strengthening epidermal cell connections and restoring skin barrier functions indicate that the application of cream containing white tea may help to close skin wounds and reduce the risk of infection. It is thought that the effects of white tea on the epidermis can be attributed to the presence of bioactive components such as polyphenols. The capacity of these components to neutralize free radicals can reduce oxidative damage, which supports cell renewal and wound healing. In addition, the reduction of inflammation signs and a faster resolution of the inflammatory response indicate the anti-inflammatory properties of the formulation. In this present study, the application of cream containing white tea to burnt skin tissue in a relatively short period of 14 days caused a tremendous improvement.

Inflammation subsequent to thermal injury represents a critical cellular phenomenon in the development of cytotoxicity [23]. The transcription factor nuclear factor-kappa B (NFk-B) is fundamentally involved in the transcriptional activation of genes that encode proinflammatory cytokines, including TNF-α and IL-1β [39,40]. A plethora of investigations have demonstrated that the inhibition of NF-κB transcriptional activity mitigates the pathological consequences that arise from inflammation following a burn injury [7,20,30]. Furthermore, reactive oxygen species (ROS) generated in the dermis subsequent to thermal injury induce structural modifications to DNA [41]. The accumulation of DNA damage and ROS triggers the activation of p53, also referred to as tumor protein 53 (TP53) [30]. The active form of p53 functions as a transcription factor that associates with DNA to form a tetrameric structure. The paramount function of p53, which regulates a multitude of genes, is to decelerate the cell cycle and initiate cellular mechanisms for DNA damage repair. In instances where the damage is irreparable, p53 orchestrates the process of apoptosis [30,42]. The presence of 8-OHdG in both nuclear and mitochondrial DNA, induced by ROS, is correlated with oxidative lesions and is consequently widely recognized as a biomarker for oxidative stress [43]. Although other nucleobases within DNA exhibit analogous reactivity with ROS, the 8-OHdG lesion is the most prevalent DNA lesion due to its relatively facile formation. Due to its relatively easy formation, the 8-OHdG lesion serves as an effective biomarker for the risk assessment of various malignancies and degenerative disorders [44].

Although the underlying mechanisms contributing to tissue damage exhibit variability contingent upon the classification of the burn, empirical studies have elucidated that such injuries precipitate lipid peroxidation, elevate the levels of malondialdehyde (MDA), diminish the availability of antioxidant enzymes (most notably, glutathione (GSH)), and engender oxidative stress through the reduction of glutathione peroxidase activity (GSH-Px) alongside an augmentation in the generation of free oxygen radicals [45,46]. Furthermore, it has been documented that ROS amplify the expression of NF-kB/p65, an established biomarker for pro-inflammatory cytokines that contribute to inflammatory processes within tissues [47]. Additionally, it has been demonstrated that ROS can elicit apoptosis in cells attributable to the heightened levels of ROS within tissues [9]. White tea (Camellia sinensis), which is characterized by the presence of EGCG and is recognized as one of the most efficacious free radical scavengers among various tea types due to its substantial content of hydroxyl groups, has been shown to mitigate oxidative stress through the inhibition of ROS production [12,48]

Oxidative stress represents a significant barrier to the reparative processes associated with tissue injuries, including wounds and burns; consequently, the mitigation of oxidative stress may facilitate the regulation of inflammation and enhance the rate of wound healing [13]. MDA, recognized as a terminal product of lipid peroxidation, serves as a biomarker for oxidative injury to cellular membranes [45,46]. Elevated levels of MDA signify an augmented state of oxidative stress and subsequent cellular injury within tissues [49]. Thus, a reduction in MDA concentrations suggests a decline in oxidative damage, thereby indicating the preservation of cellular membranes. The observed reduction in MDA levels in the current investigation implies that a cream formulated with white tea may possess efficacy in mitigating oxidative damage through the attenuation of lipid peroxidation. This phenomenon can be elucidated by the presence of potent antioxidant compounds such as polyphenols within white tea [40].

On the other hand, GSH serves as a pivotal antioxidant that is integral to cellular defense mechanisms against oxidative stress. An elevation in GSH concentrations signifies an enhancement in cellular antioxidant capacity, thereby rendering cells more resilient to oxidative stress. Antioxidants play a crucial role in safeguarding skin cells by mitigating cellular damage instigated by free radicals [50]. In the context of this investigation, the application of a cream enriched with white tea extract demonstrated a facilitative effect on the healing process and diminished the visibility of scars. This underscores the fundamental importance of antioxidants in the context of wound healing [5]. In this present research, it was noted that a wound and burn cream formulation incorporating white tea resulted in increased GSH levels and fortified antioxidant defense mechanisms, and consequently, it may exert a beneficial influence on the wound-healing trajectory. These findings imply that white tea possesses the potential to expedite wound healing through the enhancement of cellular detoxification processes, in addition to its inherent antioxidant capabilities. White tea is recognized for its abundance of antioxidant constituents, particularly polyphenols [12]. These constituents may positively influence the wound-healing process through the prevention of oxidative damage and the support of cellular defense mechanisms. Therefore, the antioxidant attributes and anti-inflammatory properties of white tea render it an appropriate candidate for facilitating the healing of burns and wounds in the integumentary system. Furthermore, the anti-inflammatory characteristics of white tea constitute a significant element in ameliorating the adverse effects of inflammation on the wound-healing process. Inflammation may enhance the healing process by aiding in the reduction of excessive edema and erythema in the localized wound region. The findings of this study indicate that the application of cream containing white tea extract substantially diminishes markers of oxidative stress and inflammation while bolstering antioxidant capacity.

In this present investigation, it was demonstrated that the cream formulation incorporating Camellia sinensis extract expedited the process of wound healing in a burn model using rats, mitigated oxidative stress and inflammation, and enhanced antioxidant capacity while concurrently reducing the expression levels of collagen I and III. Notably, despite this reduction, it was evident that the wound tissue achieved near-complete healing and exhibited a significant reduction in scar formation. These findings yield critical insights regarding the interplay between the effects of Camellia sinensis on wound healing and the regulation of the extracellular matrix (ECM) remodeling process [34]. Wound healing is a multifaceted phenomenon comprising stages of hemostasis, inflammation, proliferation, and remodeling. Throughout these stages, collagen III predominates in the initial phases of granulation tissue and contributes to the stabilization of the tissue. In the subsequent phases of the proliferation stage, collagen III is supplanted by the more resilient and robust collagen I. Nevertheless, when this process is excessive, it usually results in scar formation [3,17,51].

The formulation containing Camellia sinensis may have expedited this physiological process by mitigating inflammation and alleviating oxidative stress. The expedited wound healing may elucidate the observed reduction in collagen types I and III, as the tissue remodeling phase is accomplished with remarkable swiftness.

It has been documented in the scientific literature that inflammation promotes collagen synthesis through the modulation of fibroblast activity and the release of transforming growth factor-beta (TGF-β) [9,34]. The anti-inflammatory characteristics of Camellia sinensis may have enhanced the ECM remodeling process by attenuating these mechanisms and facilitating minimal scar formation. TGF-β serves as the principal regulator of fibroblast proliferation and collagen synthesis [9]. Nevertheless, excessive TGF-β activity may result in hypertrophic scarring and fibrosis [33]. The predominant constituent of Camellia sinensis, EGCG, modulates fibroblast activity by calibrating TGF-β signaling pathways. In this present investigation, the reduction in collagen types I and III levels may have played a significant role in the expedited wound healing and optimal remodeling of the wound tissue, attributed to this regulatory effect on TGF-β signaling.

The scarless reparative mechanisms observed in fetal wound healing may exhibit parallels to the effects attributed to Camellia sinensis. Fetal fibroblasts demonstrate differential responses to TGF-β signaling, thereby facilitating scarless healing [34]. The modulatory influence of Camellia sinensis on TGF-β signaling may have induced adult fibroblasts to exhibit behaviors akin to those of fetal fibroblasts. This phenomenon may expedite the healing process while concurrently inhibiting the formation of scars.

The potent antioxidant properties of Camellia sinensis may enhance cellular regeneration by neutralizing free radicals [9,16]. EGCG is recognized for its role in promoting epidermal regeneration through the stimulation of keratinocyte and fibroblast proliferation [7,35,52]. Such an effect may have contributed to the rapid closure of wound tissue and a reduction in the density of granulation tissue. This mechanism may facilitate the early maturation of the wound tissue and diminish the necessity for novel collagen synthesis. The observed decrease in collagen expression may represent a critical mechanism that promotes minimal scar formation.

Caspase-3 serves as an effector caspase that holds substantial significance in the mechanism of programmed cell death (apoptosis) and acts as a crucial biomarker for cellular regeneration and tissue reorganization during the process of wound healing [53]. Our findings demonstrate that the cream formulation incorporating white tea markedly decreases the positivity of caspase-3. These findings suggest the prospective advantages of white tea cream in the management of wounds and burns, indicating that it may exert a favorable influence on the wound-healing process through the modulation of apoptotic cell death. This reinforces the anti-apoptotic properties attributed to white tea extract in the context of wound and burn repair.

Apoptosis, defined as programmed cell death, serves a dual function in the modulation of wound-healing processes [54,55]. It is a critical mechanism for the elimination of damaged and dysfunctional cells during the initial phases of wound healing [56]. Additionally, apoptotic cell death facilitates the proliferation of new cells at the peripheries of the wound, constituting a vital component of the wound-healing continuum [57]. Nonetheless, the excessive or dysregulated activation of apoptosis has the potential to interfere with the healing process, thereby hindering or delaying wound repair [58,59]. Consequently, the regulation or attenuation of apoptosis may serve to expedite the wound-healing process. Oxidative stress has been identified as a trigger for apoptotic cell death; hence, the incorporation of antioxidants may exert a beneficial influence on wound healing by diminishing the rate of apoptosis. Prior investigations have demonstrated that anti-inflammatory and antioxidant agents possess the capacity to modulate apoptosis and thereby enhance the wound-healing process [13,50]. The substantial antioxidant properties of white tea may play a role in mitigating apoptotic cell death by neutralizing free radicals and curtailing the inflammatory response [11]. Antioxidant constituents may alleviate oxidative stress and inhibit apoptosis through the neutralization of free radicals. A reduction in caspase-3 activity may contribute to the preservation of healthy cells within the wound vicinity and facilitate tissue regeneration. This phenomenon may mitigate the extent of skin tissue injury and accelerate the healing trajectory, particularly in cases of burns. In this regard, the diminished caspase-3 positivity observed with the application of a cream formulation containing white tea implies that its modulatory influence on apoptosis may represent a significant mechanism underlying the wound-healing process.

The findings of this current study indicate that there exist significant disparities between the direct application of white tea to burn injuries and the utilization of a cream formulation that incorporates white tea. The direct application of white tea to the affected area yields minimal positive outcomes, whereas the cream formulation demonstrates considerable protective and restorative benefits. Several potential explanations may account for the divergent effects observed between the direct application of white tea extract and the application of a cream formulation containing white tea. Cream formulations that incorporate white tea may deliver more biocompatible and stable active compounds to the dermal surface. Furthermore, these cream formulations may facilitate a controlled release of the active constituents in white tea extract, thereby enhancing their efficacy and stability over an extended duration. This mechanism provides a sustained therapeutic effect throughout the healing trajectory, contributing to superior healing outcomes in comparison to the direct application of the extract. Additionally, cream formulations may augment the skin’s intrinsic healing capacity by safeguarding and hydrating its natural barrier. In contrast, the application of the direct extract often results in rapid evaporation or absorption by the skin, which can lead to a diminished therapeutic effect. Cream formulations may enhance the penetration of active ingredients into the deeper layers of the skin. In contrast, the direct application of the extract may remain superficial and fail to effectively infiltrate the underlying tissue layers. In conclusion, these variances likely elucidate why a cream formulation containing white tea exhibited more advantageous effects relative to the direct application of white tea extract in a rat model.

Nevertheless, prior to extrapolating the outcomes of this investigation, it is imperative to acknowledge its limitations and to assess the findings within a more comprehensive context. This research was exclusively performed utilizing a rodent model and does not derive from clinical data obtained through clinical trials involving human subjects. Initially, it is noteworthy that the rodent model does not exhibit complete congruence with human dermal characteristics; hence, direct inferences regarding human responses cannot be substantiated. Consequently, future human clinical trials are essential for a more nuanced comprehension of the implications of white tea on skin physiology. Furthermore, additional methodological enhancements are requisite to augment the precision and sensitivity of the techniques employed in this inquiry. Additionally, the influence of extraneous variables on the wound-healing mechanism may have exceeded the parameters defined by this research. Ultimately, longitudinal studies are essential to ascertain the optimal concentration, ideal dosage, and frequency of application to maximize the therapeutic efficacy of the white tea cream formulation in wound healing and to evaluate its prolonged effects. Nevertheless, it is posited that the results derived could offer a novel and efficacious therapeutic avenue within the domain of burn and wound management. The derivation of white tea extract from natural sources, along with its general safety profile, enhances the potential applicability of this cream formulation across a diverse patient population. Additionally, this study needs to be supported by studies with advanced molecular analyses addressing the mechanism of skin wound healing.

## 5. Conclusions

This present study shows that the use of a wound and burn cream containing Camellia sinensis (white tea) extract has positive effects on burn lesions and wound healing. The antioxidant and anti-inflammatory properties of white tea support the healing of skin lesions, emphasizing that it may be an effective treatment option. These findings require further investigation of the potential use of products containing white tea extract in wound and burn treatment.

## Figures and Tables

**Table 1:** Skin Histopathological Damage Score (SHDS), designed by modification of the skin damage score of Jeschke et al. and Gibson et al.

Burn Grade	Findings	Score	
-	None	0	
Grade I	Loss of keratinocytes in the Str. corneum and Str. lucidum layers	1	≤5%
		2	6–25%
		3	26–50%
		4	>50%
Grade IIa	Loss of keratinocytes in the str. corneum, str. lucidum, str. granulosum, and str. spinosum layers	1	≤5%
		2	6–25%
		3	26–50%
		4	>50%
Grade IIb	Loss of keratinocytes in the str. corneum, str. lucidum, str. granulosum, str. spinosum, and str. germinativum layers and partial lamina propria damage	1	≤5%
		2	6–25%
		3	26–50%
		4	>50%
Grade III	Loss of keratinocytes in the str. corneum, str. lucidum, str. granulosum, str. spinosum, and str. germinativum layers; lamina propria; and dermis layer damage	1	≤5%
		2	6–25%
		3	26–50%
		4	>50%
Grade IV	Loss of keratinocytes in the str. corneum, str. lucidum, str. granulosum, str. spinosum, and str. germinativum layers; lamina propria; dermis layer; muscle layer; bone and/or cartilage tissue damage	1	≤5%
		2	6–25%
		3	26–50%
		4	>50%

**Table 2:** Biochemical analysis results (mean + standard deviation).

Groups	TBARS (nmol/g Tissue)	TT (nmol/g Tissue)
Control	10.54 ± 1.11	8.03 ± 0.93
Burn	19.33 ± 0.94 ^a^	4.77 ± 0.38 ^a^
Burn + CsE	18.63 ± 0.90 ^a^	4.96 ± 0.86 ^a^
Burn + Cream	11.32 ± 1.14 ^b^	8.13 ± 1.42 ^b^

^a^ *p* < 0.001: compared with control group; ^b^ *p* < 0.001: compared with Burn group, one-way ANOVA/Tukey HSD test.

**Table 3:** Skin Histopathological Damage Scoring (SHDS) results (median (25th–75th percentiles)).

Groups	SHDS
Control	0 (0–0)
Burn	4 (4–4) ^a^
Burn + *CsE*	4 (3–4) ^a^
Burn + Cream	1 (1–1) ^b^

^a^ *p* < 0.001: compared with control group; ^b^ *p* < 0.001: compared with Burn group, Kruskal–Wallis/Tamhane T2 test.

**Table 4:** Immunopositivity scoring results in skin tissue sections stained with IHC methods (median (25th–75th percentiles)).

Group	Collagen I Immune Positivity Score	Collagen III Immune Positivity Score	Cleaved Caspase-3 Immune Positivity Score	TNF-α Immune Positivity Score	NF-Kβ/p65 Immune Positivity Score	8-OHdG Immune Positivity Score
Control	0 (0–0)	0 (0–0)	0 (0–0.5)	0 (0–0)	0 (0–0)	0 (0–0)
Burn	3 (3–4) ^a^	3 (2–3) ^a^	4 (3–4) ^a^	2 (1–2) ^a^	1 (1–2) ^a^	3 (2–3) ^a^
Burn + *CsE*	4 (3–4) ^a^	2 (2–3) ^a^	3 (2–4) ^a^	2 (1–2) ^a^	1 (1–2) ^a^	3 (2–3) ^a^
Burn + Cream	1 (1–1) ^b^	1 (1–2) ^b^	1 (1–1) ^b^	1 (0–1) ^b^	0 (0–1) ^b^	0 (0–1) ^b^

^a^ *p* < 0.001: compared with control group; ^b^ *p* < 0.001: compared with Burn group, Kruskal–Wallis/Tamhane T2 test.

## Data Availability

All data generated or analyzed during this study are included in this article. The data will be available upon reasonable request (contact: tolgamercantepe@erdogan.edu.tr).

## Supplementary Materials

The following supporting information can be downloaded at: https://www.mdpi.com/article/10.3390/life15020176/s1, Formulation of white tea (Camellia sinensis) extract cream.
