# Supplementary material for: Investigation of the Effect of Camellia Sinensis Essence Cream on Skin Burns
Source: Life (Basel). 2025 Jan 25;15(2):176. doi: 10.3390/life15020176 (PMC11856605; doi:10.3390/life15020176)
Supplement: Supplementary file 1 [file life-15-00176-s001.zip › life-3429450-supplementary.pdf]

**Compounds**	**Rate (%)**
Cetyl Stearyl	6
Jojoba oil	3
Shea oil	3
Olive oil	3
Distilled Water	70
Monopropylene glycol (MPG)	2
Glycerine	2
Tween 60	2
Tween 80	2
Vitamin E	1
Xsanthan	2
EUXYL	1
White tea leaf (*camellia sinensis*)	3

Table S1: Formulation of white tea (Camellia sinensis) extract cream.
